# Analysis of Plant Growth-Promoting Effects of Fluorescent Pseudomonas Strains Isolated from *Mentha piperita* Rhizosphere and Effects of Their Volatile Organic Compounds on Essential Oil Composition

**DOI:** 10.3389/fmicb.2016.01085

**Published:** 2016-07-19

**Authors:** Maricel V. Santoro, Pablo C. Bogino, Natalia Nocelli, Lorena del Rosario Cappellari, Walter F. Giordano, Erika Banchio

**Affiliations:** Department of Biología Molecular, Facultad de Ciencias Exactas, Químicas y Naturales, Universidad Nacional de Río CuartoRío Cuarto, Argentina

**Keywords:** *Mentha*, fluorescent *Pseudomonas*, genotyping, ARDRA, essential oils, microbial volatile organic compounds

## Abstract

Many species or strains of the genus Pseudomonas have been characterized as plant growth promoting rhizobacteria (PGPR). We used a combination of phenotypic and genotypic techniques to analyze the community of fluorescent Pseudomonas strains in the rhizosphere of commercially grown *Mentha piperita* (peppermint). Biochemical techniques, Amplified rDNA Restriction Analysis (ARDRA), and 16S rRNA gene sequence analysis revealed that the majority of the isolated native fluorescent strains were *P. putida*. Use of two Repetitive Sequence-based PCR (rep-PCR) techniques, BOX-PCR and ERIC-PCR, allowed us to evaluate diversity among the native strains and to more effectively distinguish among them. PGPR activity was tested for the native strains and reference strain *P. fluorescens* WCS417r. Micropropagated *M. piperita* plantlets were exposed to microbial volatile organic compounds (mVOCs) emitted by the bacterial strains, and plant biomass parameters and production of essential oils (EOs) were measured. mVOCs from 11 of the native strains caused an increase in shoot fresh weight. mVOCs from three native strains (SJ04, SJ25, SJ48) induced changes in *M. pierita* EO composition. The mVOCs caused a reduction of metabolites in the monoterpene pathway, for example menthofuran, and an increase in menthol production. Menthol production is the primary indicator of EO quality. The mVOCs produced by native strains SJ04, SJ25, SJ48, and strain WCS417r were analyzed. The obtained mVOC chromatographic profiles were unique for each of the three native strains analyzed, containing varying hydrocarbon, aromatic, and alogenic compounds. The differential effects of the strains were most likely due to the specific mixtures of mVOCs emitted by each strain, suggesting a synergistic effect occurs among the compounds present.

## Introduction

Peppermint (*Mentha piperita* L.; family *Labiatae*) is a popular, commonly-used flavoring agent worldwide. Fresh or dried leaves of *Mentha* species are used primarily as condiments. Essential oils (EOs) of these plants, which are produced and stored in glandular hairs, are used as food and beverage flavorings, as fragrances, and as fungicides or insecticides in a variety of pharmaceutical and industrial products (Harrewijn et al., 2001; Ram et al., 2006). EOs are secondary metabolites whose production is associated with primary metabolism and with availability of soil nutrients (Shulka et al., 1992).

Among the many known PGPR genera, *Pseudomonas* has received the most research attention because it is widely distributed in various environments and is easy to culture under laboratory conditions (Palleroni, 2005). Members of the “fluorescent *Pseudomonas* group” produce a water-soluble fluorescent green-yellow pigment termed pseudobactin or pyoverdine (Stanier et al., 1966). This characteristic peptide displays iron-binding activity and is associated with pathogenic or growth-promoting properties (Fuchs et al., 2001; Mehri et al., 2011). Fluorescent *Pseudomonas* strains have been isolated from rhizospheric soils of numerouscropplants, including cotton (Hu et al., 2005), rice (Loaces et al., 2011), banana (Naik et al., 2008), rape (Patten and Glick, 2002), sugar cane (Rameshkumar et al., 2012), wheat, and barley (Mavrodi et al., 2001; Parejko et al., 2012). The two major fluorescent *Pseudomonas* species that display PGPR activity are *P. fluorescens* and *P. putida* (Kloepper and Schroth, 1978). Few studies to date have focused on PGPR isolated from rhizospheres of aromatic plant species because of the presumption that EOs may be released in root exudates and display antimicrobial activity (Chen et al., 2004). We used phenotyping and genotyping techniques to isolate, identify, and characterize fluorescent *Pseudomonas* strains from *M. piperita* rhizosphere of *M. piperita*. We then evaluated PGPR activity of these strains on micropropagated *M. piperita* plants, including mVOC-mediated effects on plant biomass and EO production.

## Materials and methods

### Sample collection and strain isolation

A total of 20 *M. piperita* plants were collected randomly from a commercial crop (San José) grown in the San Javier department, Córdoba province, Argentina (30°30′00.0″S, 64°30′00.0″W). Samples were placed individually in plastic bags, stored at 4°C, transported and processed within 24 h. Five roots were washed in sterile distilled water to remove loosely adherent soil, and then rotary shaken 15 min in 100 ml sterile phosphate buffer, pH 7.5, to obtain a rhizospheric soil suspension. Serial dilutions were prepared from the suspension, and 0.1 ml of each dilution was spread on Nutrient Agar medium (Laboratorios Britania, Buenos Aires, Argentina) and on King B medium (King et al., 1954) and incubated at 28°C for 48 h. After complete evaporation of buffer from soil, total bacteria and fluorescent bacteria under UV light were counted as colony forming units (CFU) per g rhizospheric soil (Mehnaz et al., 2009). Bacterial strains were purified on King B medium, identified, and characterized. Fifty fluorescent strains were isolated (Table 1), and cryopreserved at −80°C in 20% glycerol (v/v). A single colony from each strain was grown aerobically on LB medium (Luria and Burrous, 1955) and incubated on a rotary shaker (200 rpm) for 24 h at 28°C prior to testing. This isolation from roots was performed four times.

**Table 1:** **Phenotypic characterization of fluorescent ***Pseudomonas*** strains, based on morphological, physiological, and biochemical evaluation of native strains isolated from the ***M. piperita*** rhizosphere**.

	**Colony morphology**	**Gram**	**Catalase**	**Oxidase**	**UV Fluorescence**	**Pigmentation**		**Growth**				**Hydrolysis**				
						**King a**	**King b**	**4°C**	**42°C**	**Nacl 5%**	**Nacl 6.5%**	**Tween 80**	**Starch**	**Gelatin**	**Casein**	**Lecithinase**
SJ1	Smooth and gleaming	−	+	+	Green	−	Green	−	±	+	−	−	−	−	−	−
SJ4	Smooth and gleaming	−	+	−	+	−	−	+	±	−	+	−	nd	nd	−	−
SJ7b	Smooth and gleaming	−	+	+	−	Orange	Orange	+	−	+	−	+	−	−	+	−
SJ8	Smooth and gleaming	−	+	+	−	nd	Orange	±	nd	+	−	−	−	−	−	−
SJ9	Smooth and gleaming	−	+	+	Light blue	−	Green	−	±	+	+	−	−	−	−	−
SJ10	Smooth and gleaming	−	+	+	Green	−	Green	−	±	+	+	−	−	−	−	−
SJ11	Smooth and gleaming	−	+	+	Green	−	Green	−	±	+	−	−	−	−	−	−
SJ12	Smooth and gleaming	−	+	+	Light blue	−	−	−	±	+	−	−	−	−	−	−
SJ13	Smooth and gleaming	−	+	+	Light blue	−	Green	−	±	+	+	−	−	−	−	−
SJ16	Smooth and gleaming	−	+	+	+	−	−	−	−	+	+	−	−	−	−	−
SJ17	Smooth and gleaming	−	+	+	Green	−	Green	−	+	+	−	−	−	−	−	−
SJ18	Smooth and gleaming	−	+	+	Green	−	Green	−	+	+	−	−	−	−	−	−
SJ20	Smooth and gleaming	−	+	+	+	−	−	−	−	+	+	−	−	−	−	−
SJ21	Smooth and gleaming	−	+	+	Green	−	Green	−	−	+	−	−	−	−	−	−
SJ22	Smooth and gleaming	−	+	+	Green	−	Green	−	−	+	+	−	−	−	−	−
SJ24	Smooth and gleaming	−	+	+	+	−	−	−	−	+	+	−	−	−	−	−
SJ25	Smooth and gleaming	−	+	+	Green	−	Green	−	−	+	+	−	−	−	−	−
SJ27	Smooth and gleaming	−	+	+	Green	−	Green	−	−	+	−	−	−	−	−	−
SJ28	Smooth and gleaming	−	+	+	+	−	−	−	−	+	+	−	−	−	−	−
SJ29	Smooth and gleaming	−	+	+	Green	−	Green	−	−	+	−	−	−	−	−	−
SJ30	Smooth and gleaming	−	+	+	Light blue	−	Green	−	−	+	+	−	−	−	−	−
SJ31	Smooth and gleaming	−	+	+	Green	−	Green	−	−	+	−	−	−	−	−	−
SJ32	Smooth and gleaming	−	+	+	Green	−	Green	−	−	+	−	−	−	−	−	−
SJ33	Smooth and gleaming	−	+	+	Green	nd	−	−	nd	+	−	−	−	−	−	−
SJ34	Smooth and gleaming	−	+	−	Green	−	−	+	−	+	−	+	−	+	+	+
SJ35	Smooth and gleaming	−	+	+	Green	−	Green	−	−	+	−	−	−	−	−	−
SJ36	Smooth and gleaming	−	+	+	Light blue	−	−	−	−	+	−	−	−	−	−	−
SJ37	Smooth and gleaming	−	+	+	Green	−	Green	−	−	+	−	−	−	−	−	−
SJ38	Smooth and gleaming	−	+	+	Green	−	Green	−	−	+	−	−	−	−	−	−
SJ39	Smooth and gleaming	−	+	+	Green	−	Green	−	−	+	−	−	−	−	−	−
SJ40	Smooth and gleaming	−	+	+	Green	−	Green	−	−	+	−	−	−	−	−	−
SJ41	Smooth and gleaming	−	+	+		−	−	−	−	+	+	−	−	−	−	−
SJ42	Smooth and gleaming	−	+	+	Green	−	Green	−	−	+	−	−	−	−	−	−
SJ44	Smooth and gleaming	−	+	+	Green	−	Green	−	−	+	+	−	−	−	−	−
SJ45	Smooth and gleaming	−	+	+	Green	−	−	±	−	+	−	−	−	−	−	−
SJ46	Smooth and gleaming	−	+	−	−	nd	−	+	nd	+	−	−	−	−	−	−
SJ47	Smooth and gleaming	−	+	+	Green	−	Green	−	−	+	+	−	−	−	−	−
SJ48	Smooth and gleaming	−	+	+	Green	−	Green	−	±	+	−	−	−	−	−	−
PAO I	Smooth and gleaming	−	+	+	Green	Blue	Green	−	++	+	+	+	−	+	+	+
KT2440	Smooth and gleaming	−	+	+	Green	−	Green	+	−	+	±	−	−	−	−	−
WCS417r	Smooth and gleaming	−	+	+	Green	−	Green	+	−	+	−	+	−	−	+	+

### Analysis of EO components in root exudates

**Figure 1:**
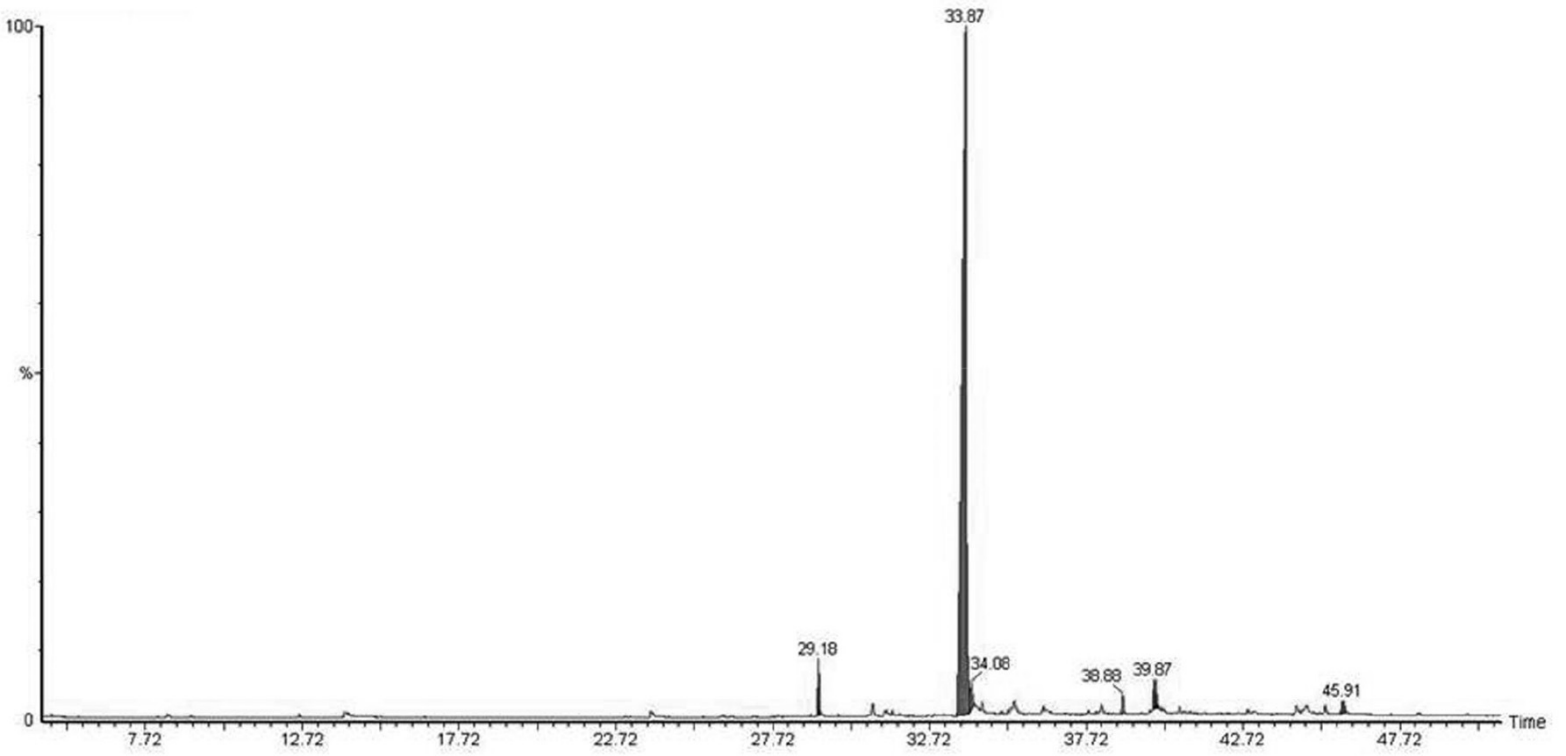
**GC profile of ***M. piperita*** root exudates**.

Micropropagated *M. piperita* plantlets were grown on MS liquid medium (Santoro et al., 2011) for 30 days. The medium was then filtered (pore diameter 0.46 μm), and the volatile fraction was extracted with dichloromethane. EOs were analyzed and detected by gas chromatography/mass spectrometry (GC-MS; Figure 1; Table 2). Flame ionization detector (FID) response factors for each compound generated essentially equal areas (differences<5%). Chemical analyses were performed by GC (model Clarus 600, PerkinElmer; Waltham, MA, USA) with a CBP-1 capillary column (30 m × 0.25 mm, film thickness 0.25 mm) and mass-selective detector. Analytical conditions: injector temperature 250°C, detector temperature 270°C; oven temperature programmed from 60°C (3 min) to 240°C at 4°C min^−1^; carrier gas ¼ helium at constant flow 0.9 ml min^−1^; source 70 eV. EO components were identified based on mass spectra and retention times, in comparison with standards.

**Table 2:** **Identification and relative percentages of major chemical signals detected in ***M. piperita*** root exudates, based on GC-MS analysis and comparison with the NIST database, with acceptance of SI ≥ 800**.

**Retention time**	**Relative percentage**	**Identification**
29.183	2.31	3,4-dihydro-2H-1,5-(3″-t-butyl)benzodioxepine
33.870	90.82	4-octylbutan-4-olide
34.075	1.90	2(3H)-furanone, 5-heptyldihydro-
38.877	0.79	7,9-di-tert-butyl-1-oxaspiro(4,5)deca-6,9-diene-2,8-dione
39.873	1.35	1,2-benzenedicarboxylic acid, dibutyl ester
39.948	1.63	n-hexadecanoic acid
45.910	1.21	Z-2-octadecen-1-ol

### Phenotypic characterization

Fluorescent bacteria were phenotypically characterized based on morphological, physiological, and biochemical criteria (Lysenko, 1961; Stanier et al., 1966; Palleroni, 2005; Table 1). The tests and parameters included colony morphology, Gram determination, catalase determination (Urzí et al., 2001), oxidase determination (kit fromLaboratorios Britania), fluorescence color under UV illumination, pigment production (King et al., 1954), temperature tolerance, growth at 5 and 6.5% salt concentration, gelatin hydrolysis (Medina and Baresi, 2007), Tween 80 hydrolysis (Sierra, 1957), starch hydrolysis (Gordon and Mihm, 1956), casein hydrolysis (Salisbury and Lykos, 1972), and lecithinase production (Sneath, 1956). Each test was performed twice.

### DNA extraction

A single colony was inoculated on 3 ml LB medium and incubated at 28°C for 24 h. A 2-ml culture sample taken at late exponential growth phase was subjected to DNA extraction using a Genomic DNA Purification Kit (Fermentas, Thermo Fisher Scientific; Pittsburgh, PA, USA) according to the manufacturer's instructions.

### Genotypic testing

#### Amplified ribosomal DNA restriction analysis (ARDRA)

*16S rRNA* gene amplification was performed, primers and methodology are specified in Table 3. A single specific band (~1400 bp) was observed by UV transillumination, and endonuclease digestion analysis of each amplicon was performed separately with *Msp*I, *Hinf* I, *Hae*III, and *Alu*I (Achouak et al., 2000; Wu et al., 2009; Mehri et al., 2011; Nievas et al., 2012) according to the manufacturer's (Fermentas') instructions. Reaction mixtures were incubated overnight at 37°C. Total volume of each restriction-digested product was evaluated by 3% (w/v) agarose-ethidium bromide stained gel electrophoresis and photographed for subsequent analysis.

**Table 3:** **Primers sequences used for genotypic analysis of native fluorescent ***Pseudomonas*** and reference strains**.

**Genotipic test**	**Primers**	**References**	**Methodology**
ARDRA	fD1 (5′-AGAGTTTGATCCTGGCTCAG-3′) rD1 (5′-AAGGAGGTGATCCAGCC-3′)	Weisburg et al., 1991	Nievas et al., 2012
*16S rRNA* sequencing	27F (5′-AGAGTTTGATCCTGGCTCAG-3′) 1492R (5′-TACGGTTACCTTGTTACGACTT-3′)	Willems et al., 2001	Willems et al., 2001
ERIC-PCR	E1 (5′-ATGTAAGCTCCTGGGGATTCAC-3′) E2 (5′-AAGTAAGTGACTGGGGTGACGG-3′)	Versalovic et al., 1991.	Nievas et al., 2012
BOX-PCR	BOX1AR (5′- CTACGGCAAGGCGACGCTGACG- 3′)	Versalovic et al., 1994	Wolska et al., 2011

#### *16S rRNA* gene nucleotide sequence analysis

Nucleotide sequence analysis of the *16S rRNA* gene was performed for 17 native strains (Table 4). Direct PCR was performed with 1 μl DNA template in a 20-μl PCR reaction mixture using primer and methodology specified in Table 3. Purified PCR products (~1400 bp) were sequenced by Macrogen, Inc. (Seoul, Korea) using an automated DNA sequencing system (model 3730XL, Applied Biosystems; Foster City, CA, USA). Sequence identities were determined by BLAST search program (National Center for Biotechnology Information [NCBI]; Bethesda, MD, USA; Altschul et al., 1997). Nucleotide sequences of the *16S rRNA* gene for each analyzed strain were deposited in GenBank with respective accession numbers (Table 5).

**Table 4:** **RFLP patterns of ***16SrRNA*** genes of native and reference strains of fluorescent ***Pseudomonas*****.

	***16S rRNA* genotype^a^**	**RFLP pattern^b^**			
**Strain**		***Hinf*I**	***Msp*I**	***Hae*III**	***Alu*I**
SJ01	2	A	B	B	B
SJ04	6	B	A	A	C
SJ7b	6	B	A	A	C
SJ08	7	B	B	B	D
SJ09	3	A	A	A	C
SJ10	3	A	A	A	C
SJ11	10	A	B	D	B
SJ12	15	D	B	C	E
SJ13	1	A	A	A	A
SJ16	2	A	B	B	B
SJ17	4	A	A	B	A
SJ18	2	A	B	B	B
SJ20	2	A	B	B	B
SJ21	13	C	B	B	A
SJ22	1	A	A	A	A
SJ24	1	A	A	A	A
SJ25	9	A	B	A	A
SJ27	1	A	A	A	A
SJ28	4	A	A	B	A
SJ29	1	A	A	A	A
SJ30	1	A	A	A	A
SJ31	1	A	A	A	A
SJ32	1	A	A	A	A
SJ33	1	A	A	A	A
SJ34	11	B	B	B	B
SJ35	1	A	A	A	A
SJ36	1	A	A	A	A
SJ37	1	A	A	A	A
SJ38	14	C	A	A	A
SJ39	8	C	B	B	B
SJ40	8	C	B	B	B
SJ41	5	A	B	B	A
SJ42	1	A	A	A	A
SJ44	5	A	B	B	A
SJ45	1	A	A	A	A
SJ46	7	B	B	B	D
SJ47	1	A	A	A	A
SJ48	1	A	A	A	A
WCS417r	12	B	A	B	F
KT2440	3	A	A	A	C

a *The 16S rRNA genotype represents a combination of RFLP patterns obtained from four restriction enzymes*.

b *Strain with the same letter have the same RFLP pattern obtained from one endonuclease*.

**Table 5:** **BLAST analysis of ***16SrRNA*** gene sequences of native strains isolated from ***M. piperita*** rhizosphere**.

**Strain**	**ARDRA analysis**			***16SrRNA*** **analysis**	
	**Genotype**	**Group**	**Accession number**	**Significant Alignment**	**Identity (%)**
SJ01	2	B	KF312466.1	*P. putida* C-G-PDA3 – HM755498.1	99
SJ04	6	A	KF312467.1	*P. putida* GM6 – DQ133506.1	99
SJ7b	6	A	KF312468.1	*P. fluorescens* MazP22 – JX885768.1	99
SJ08	7	B	KF312469.1	*Pseudomonas* sp.P97.38 – DQ453823.1	99
SJ13	1	A	KF312470.1	*P. putida* NBRC102090 – AB681701.1	99
SJ16	2	B	KF312471.1	*P. putida* S18 – AY741157.1	99
SJ17	4	A	KF312472.1	*Pseudomonas* sp. MKP213 – KC013979.1	99
SJ25	9	B	KF312473.1	*Pseudomonas* sp. MKP213 – KC013979.1	99
SJ28	4	A	KF312474.1	*P. putida* 1599 – JN679860.1	99
SJ31	1	A	KF312475.1	*Pseudomonas* sp. MKP213 – KC013979.1	99
SJ32	1	A	KF312476.1	*Pseudomonas* sp. ONBA-17 – DQ079062.1	99
SJ45	1	A	KF312477.1	Pseudomonas sp. m4(2012) – JX675235.1	99
SJ46	7	B	KF312478.1	Ps. fluorescens Pf29A – DQ473439.1	99
SJ48	1	A	KF312479.1	*P. putida* NBRC102090 – AB681701.1	99

**Figure 2:**
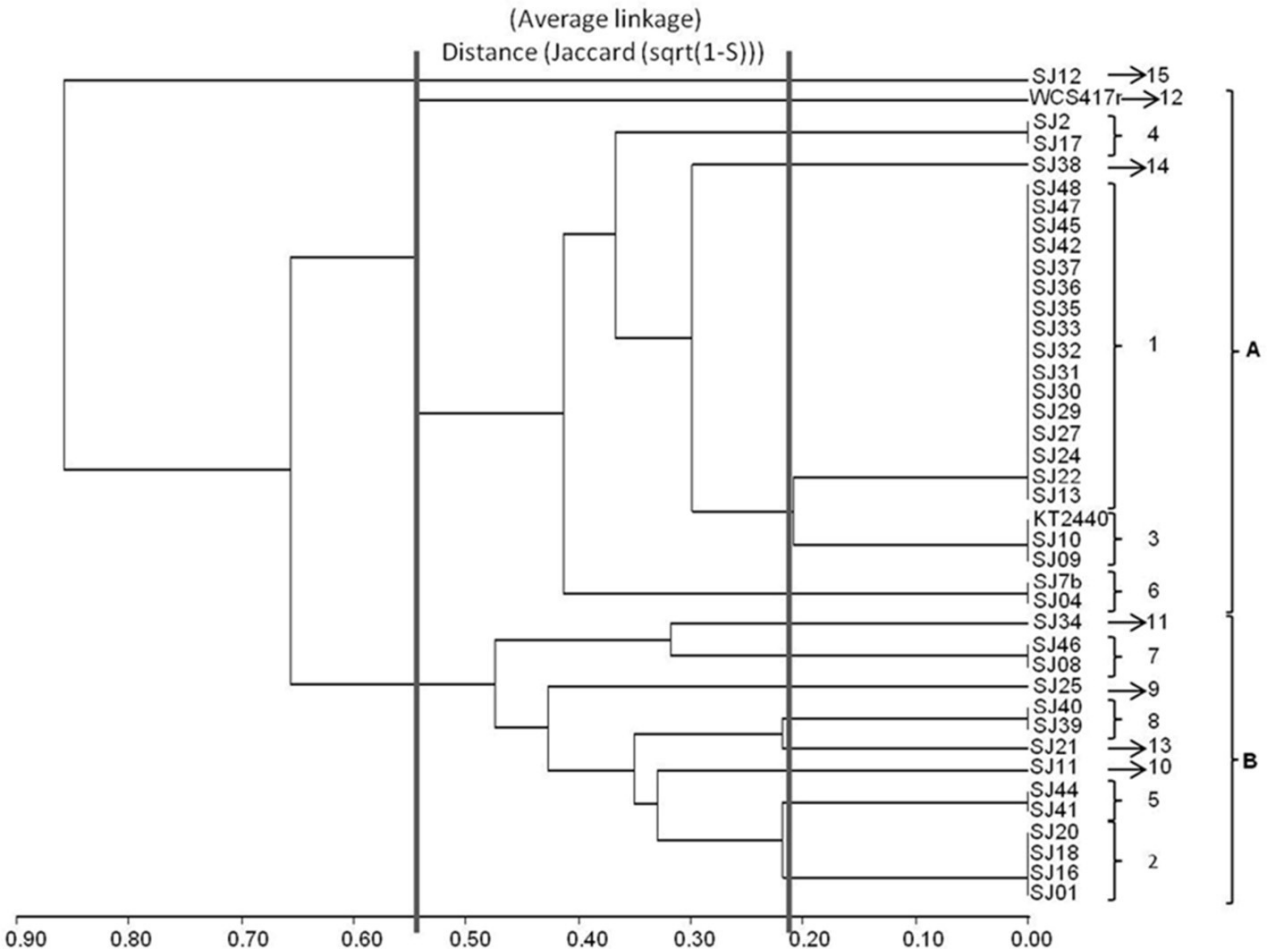
**Dendrogram generated from RFLP patterns of ***16S rRNA*** genes of native and reference strains of fluorescent ***Pseudomonas***, using the UPGMA algorithm and Jaccard coefficient**.

To generate a phylogenetic tree reflecting evolutionary relationships among fluorescent *Pseudomonas* strains, phylogenetic analysis was performed using the MEGA 4.0.2 software program (Tamura et al., 2007). Multiple alignments were made with the Clustal W software program (Higgins et al., 1994). Aligned sequences were used to construct a phylogenetic tree based on the neighbor-joining method (Saitou and Nei, 1987), using the Kimura two-parameter model (Kimura, 1980; Figure 2).

#### ERIC-PCR fingerprinting

Enterobacterial Repetitive Intergenic Consensus (ERIC) fragments were analyzed for 40 native fluorescent strains. Amplification was performed using primers and methodology specified in Table 3. PCR products were separated by 1.2% (w/v) agarose gel electrophoresis in TAE 1X buffer, stained with ethidium bromide, and photographed for subsequent analysis.

#### BOX-PCR fingerprinting

Repetitive extragenic palindromic sequences (BOX-PCR) were evaluated for the 40 strains. Amplification was performed with primer and methodology specified in Table 3. Amplified products were loaded on 2% (w/v) agarose gel electrophoresis, stained with ethidium bromide, and run at 60 V for 30 min and90 V for 120 min. The obtained gel was photographed for latter analysis.

#### Analysis of genotypic data

Fingerprints generated by ARDRA and by Repetitive Sequence-based PCR (rep-PCR) techniques (ERIC-PCR, BOX-PCR) as above were analyzed manually. Presence or absence of each band in the PCR profile was coded as a binary matrix. Genetic similarity and clustering analysis for each generated matrix were performed using the Infogen software program, v. 2011 (Universidad Nacional de Córdoba, Argentina). Genetic distances among native fluorescent strains were evaluated using a Jaccard coefficient. Clustering was visualized by construction of dendrograms using the Unweighted Pair-Group Method with Arithmetic Average (UPGMA) algorithm (Di Renzo et al., 2001; Balzarini, 2003).

### Evaluation of growth-promoting effects

#### Bacterial suspension

Strains were grown aerobically on LB liquid medium for 24 h at 30°C with rotary shaking. Saturated cultures were diluted in sterile distilled water to final concentration 10^9^ CFU ml^−1^.

#### Exposure to mVOCs

Plastic Petri dishes (90 × 15 mm) with a center partition (I-plate, Fisher Scientific) were used. One side of the dish contained MS solid medium, and the other contained Hoagland medium (Hoagland and Arnon, 1938) added with 3% sucrose (w/v) and 1.5% agar (w/v). Bacterial suspension as above (one drop; 20 μl) was placed on the Hoagland side. Distilled water (one drop) was used as negative control. A single node from a young shoot of aseptically cultured *M. piperita* (Santoro et al., 2011) was placed on the opposite side of the partition. In this way, plants were exposed to mVOCs without physical contact. Dishes were parafilm-sealed, arranged in a completely randomized design, and placed in a growth chamber under controlled conditions of light (16/8-h light/dark cycle), temperature (22 ± 2°C), and relative humidity (~70%). Five plants were used for each treatment, and experiments were replicated four times.

#### Plant biomass and morphology

Plants exposed to mVOCs were harvested after 30 days and evaluated for shoot length, root length, shoot fresh weight, root dry weight, node number, leaf number, and shoot ramification number (Santoro et al., 2011).

#### Extraction and analysis of EOs

Each shoot sample was weighed, subjected to hydrodistillation in a micro Clevenger-like apparatus for 14 min, and the volatile fraction was extracted with dichloromethane. An internal standard (1 μl γ-dodecalactone in 100 μl ethanol) was added during the distillation procedure. Oil components were initially identified based on mass spectral and retention time data and confirmed by direct comparison with commercial standards (Sigma-Aldrich). FID response factors for each compound generated essentially equal areas (differences <5%). For comparison of results under different treatments, response factors for individual compounds were assumed to be equal (Shukui et al., 2005). Chemical analyses of EOs were performed as described in Section Analysis of EO Components in Root Exudates. For each plant, areas corresponding to analyzed compounds were added, and total weight of EOs from each sample was expressed relative to area of the internal standard. Shoots were weighed, and yield was expressed as μg g^−1^ fresh weight.

#### Statistical analysis

Data were subjected to analysis of variance (ANOVA) followed by comparison of multiple treatment levels with control, using post hoc Fisher's Least Significant Difference (LSD) test. Infostat software program, v. 2.0 (Group Infostat, Universidad Nacional de Córdoba, Argentina) was used for all statistical analyses.

### Chemical analysis of mVOCs

#### mVOC collection

The three bacterial strains that had the greatest effect on plant biomass and EO production (SJ04, SJ25, SJ48) were cultured for mVOC analysis, and WCS417r was included as reference strain. Bacteria were cultured in sealed 100-ml Kitasato flasksin Hoagland medium plus 0.4% TSB for 48 h at 30°C. The flasks were then connected to a vacuum pump to guarantee air flow, and the flaskinlet was covered with a cap that had been pre-drilled and modified with a cotton-filled glass tube to control air flux to the collection trap. Air exited the chamber through a reusable glass collection trap packed with 30 mg Super-Q absorbent (80–100 mesh; Alltech Assoc., Deerfield, IL, USA) to capture mVOCs. Prior to mVOC collection, eachfilter was rinsed with 2 ml dichloromethane and 2 ml hexaneto remove impurities. Flasks were then placed on a heating block at 50°C with magnetic stirring for 30 min. The filters were immediately eluted from absorbent traps with 200 μl dichloromethane, and collected mVOCs were analyzed by GC-MS as described below.

#### GC-MS analysis and conditions

Filter samples were concentrated with nitrogen gas, resuspended in ethanol, and injected into a Perkin-Elmer Clarus600 gas chromatograph equipped with DB5 column (60 m, i.d. 0.25 mm, film thickness 0.25 μm; J&W Scientific, Folsom, CA, USA) and mass-selective detector. GC-MS conditions: 25-minruns; injection port operated in splitless mode with constant He flow 1.0 ml/min; initial oven temperature 33°C, held for 3 min, ramped at 10°C min^−1^ to 180°C, ramped at40 min^−1^ to 220°C, and held for 5 min; HP quadrupole mass spectrometer operated in electron ionization mode at 70 eV; source temperature 200°C; quadrupole temperature 150°C; continuous scan from m/z 40 to 500. Volatile components were identified by comparison of retention times and recorded mass spectra against the NIST database, with acceptance of similarity index (SI) ≥ 800.

## Results and discussion

### Sample and strain isolation

*Pseudomonas* is the most extensively studied of the known PGPR genera because the species are widely distributed in many different environments and are easy to culture under laboratory conditions. For isolation of fluorescent *Pseudomonas* strains, rhizospheric soil from a commercial *M. piperita* crop as described in Section Sample Collection and Strain Isolation was collected, with total bacteria count 3.05 × 10^7^ CFU g^−1^. Similar results were obtained in previous studies of rhizospheric soil from other crops (Torsvik et al., 1996; Houlden et al., 2008; Mehnaz et al., 2009). In total 4.7 × 10^5^ CFU g^−1^ fluorescent bacteria were counted, corresponding to 1.54% of total bacteria. Fifty colony isolates were collected based on observation of fluorescence under UV light (Table 1).

Various types of soil used for crop production support highly diverse microbial communities, and introduction of crop plants affects those communities by promoting or suppressing growth of indigenous bacteria (Houlden et al., 2008; Agaras et al., 2012). Whether the primary determinant of microbial community structure is soil properties or introduced plants remains controversial. Plants clearly have a substantial effect on microbial communities based on the composition of their root exudates, which is closely associated with nutritional status and growth stage (Houlden et al., 2008). The proportion of fluorescent bacteria relative to total count of culturable bacteria in rhizospheric soil or bulk soil has not been addressed in previous studies. Agaras et al. (2012) reported that fluorescent strains accounted for 10–94% of total *Pseudomonas* in various crop soils and bulk soils. Results of the present study suggest that selection by *M. piperita* root exudates may explain the much lower count (~10^7^ CFU g^−1^) of culturable bacteria for this species than for other crop plants (~10^10^ CFU g^−1^; Agaras et al., 2012; Bogino et al., 2013).

### Analysis of root exudates

Native bacterial strains have not been isolated from the rhizosphere of aromatic crop plants in previous studies, because EOs present in root exudates are generally presumed to have a toxic effect on microorganisms (Chen et al., 2004). We analyzed root exudates from *in vitro* cultured *M. piperita* by GC-MS (Figure 1), and evaluated fragmentation patterns by comparison with the NIST database, with acceptance of SI ≥ 800 (Table 2). No monoterpenes were detected in the root exudates, indicating that bacterial communities can be established in the *M. piperita* rhizosphere. The absence of EOs in root exudates suggests that the low total bacterial count did not reflect selection based on plant antimicrobial properties, e.g., inhibition or suppression of bacterial growth. Rather, the low count may result from competition or inhibition of established communities through direct growth inhibition by microbial compounds, or from adaptation by bacteria to the soil environment, edaphic conditions, and/or altitude.

### Phenotypic testing

All 50 isolated strains were subjected to phenotypic testing. Three strains representing the main groups of fluorescent *Pseudomonas* were used as references: *P. fluorescens* WCS417r, *P. putida* KT2440, and *P. aeruginosa* PAO I (Lysenko, 1961; Stanier et al., 1966; Palleroni, 2005). Biochemical characteristics of the isolated strains are summarized in Table 1. In terms of biochemical responses, 33 strains (66% of total) were most similar to *P. putida* KT2440, one (SJ34) was similar to *P. fluorescens* WCS417R, and none were similar to *P. aeruginosa* PAO I. The 50 strains were subjected to genotypic testing (Sec. 3.4) for identification of *Pseudomonas* species, and screened for PGPR activity mediated by production of mVOCs (Section Plant Growth-Promoting Effects of mVOCs). Strains SJ04, SJ7b, and SJ46 were included in the genotypic testing because they showed the strongest promoting effects on biomass and EO production of micropropagated *M. piperita* plants.

### Genotypic testing

Three molecular techniques (ARDRA, ERIC-PCR fingerprinting, BOX-PCR fingerprinting) were applied to evaluate similarities through clustering analysis of the isolated fluorescent *Pseudomonas* strains. Forty native strains and two reference strains (*P. fluorescens* WCS417r, *P. putida* KT2440) were evaluated. *P. aeruginosa* PAO I was excluded because none of the native strains showed biochemical similarity to it.

#### ARDRA

The ARDRA technique (analysis of16S rRNA gene fragments produced by digestion with restriction enzymes) has been widely applied in genetic diversity studies of Pseudomonas and other rhizobacteria. It has been used successfully for analysis of fluorescent Pseudomonas communities isolated from rhizospheres of various crop plants under a variety of environmental and climatic conditions (Prosser, 2002; Mehri et al., 2011; Nievas et al., 2012). It has also been used to study variation of Pseudomonas communities as a function of crop management strategies (Achouak et al., 2000) and application of pesticides or other chemical compounds (Viti and Giovannetti, 2005; Braun et al., 2006; Wang et al., 2008; Wu et al., 2009). The present study is the first to apply ARDRA to PGPR isolates from the *M. piperita* rhizosphere.

We used restriction endonucleases *Alu* I, *Msp* I, *Hinf* I, and *Hae* III because of their ability to discriminate among *Pseudomonas* strains (Achouak et al., 2000; Wu et al., 2009; Mehri et al., 2011). For each analyzed strain, a single amplicon (~1400 bp) was obtained using the primers rD1 and fD1. Only seven of the 40 markers gave monomorphic bands. *Alu*I had the highest polymorphism, generating six different Restriction Fragment Length Polymorphism (RFLP) patterns. *Hinf* I and *Hae* III each generated four RFLP patterns, and *Msp*I generated two RFLP patterns, resulting in less informative restriction (Table 4). *16S rRNA* RFLP patterns obtained using each of the endonucleases were combined to obtain a general genotype for each strain. Genotype 1, the most frequent, was present in 16 native fluorescent strains. The genotype of *P. putida* KT2440 was also found in two other strains, while the genotype of *P. fluorescens*WCS417r was unique (Table 4).

A dendrogram was constructed as described in Section Analysis of Genotypic Data to determine genetic distances among native *Pseudomonas* strains (Figure 2). At 35% similarity, the dendrogram showed a division into two major clusters (A and B). At 45% similarity, cluster A grouped 25 strains having genotypes 1, 3 (including *P. putida* KT2440), 4, 6, 12 (including *P. fluorescens* WCS417r), and 14. Within cluster A, genotypes 1 and 3 were highly related (80%similarity). Cluster B grouped the remaining strains at 35% similarity. These strains had genotypes 2, 5, 7, 8, 9, 10, and 13. Within cluster B, genotypes 8 and 13, and 5 and 2 were highly related (80% similarity).

The genotypic analysis revealed high correlations between *P. putida* KT2440 and ≥ 50% of total fluorescent strains isolated from the *M. piperita* rhizosphere, consistent with the results of biochemical analysis. Strains with genotype 7 differed from *P. putida* KT2440 in both genotypic and phenotypic analyses. Strains with genotype 10, 2, 13, 9, 8, and 5 displayed genotypic divergence from *P. putida* KT2440 in spite of phenotypic similarities. Similar divergence was observed between strains with genotype 11 and *P. fluorescens* WCS417r (Table 4; Figure 2).

In a comparison of the phenotypic and genotypic results, clustering discrepancies may result from the differing characteristics evaluated by the methodologies. Phenotypic testing detects metabolic activities that may be shared among various species within a particular genus, whereas genotypic testing detects similarities or distances among genetic markers generated from a defined gene. Regardless of these considerations, 60% of native fluorescent rhizobacterial strains displayed high biochemical and genetic similarity with *P. putida* KT2440, and 50% of strains showed 80% genotypic similarity with KT2440. *P. putida* is clearly the major fluorescent strain present in the *M. piperita* rhizosphere.

Results have shown that the*16SrRNA* gene is highly conserved, causing difficulty in differentiating among closely related species. It is therefore preferable to apply two or more molecular techniques. Inclusion of techniques that identify sequences repeated along the whole genome permits analysis of more data, and of differences in sequences of more than one gene (Grimont, 2002; Rangel-Castro et al., 2002; Gomila et al., 2007; Naik et al., 2008; Mehri et al., 2011).

#### *16S rRNA* gene nucleotide sequence analysis

The analyzed strains were selected based on their abundance of genotypes and distribution in clusters A and B (Table 4). PGPR activity of strains on micropropagated *M. piperita* plantlets mediated by mVOC production (Section Plant Growth-Promoting Effects of mVOCs) was also taken into consideration.

The BLAST software program was used to analyze the obtained *16S rRNA* gene sequences and to establish their alignment and identity with reference strains (Altschul et al., 1997). All of the analyzed strains had identities within the genus *Pseudomonas*. The native strains had high percentage identity with *P. putida* and *P. fluorescens*. Strains SJ13 and SJ48, and SJ17 and SJ25,had the same identity (Table 5). They should be considered different strains of the same species, in view of their very low similarity in ERIC-PCR and BOX-PCR analyses (Sections ERIC-PCR, BOX-PCR), which deeply probe strain diversity.

**Figure 3:**
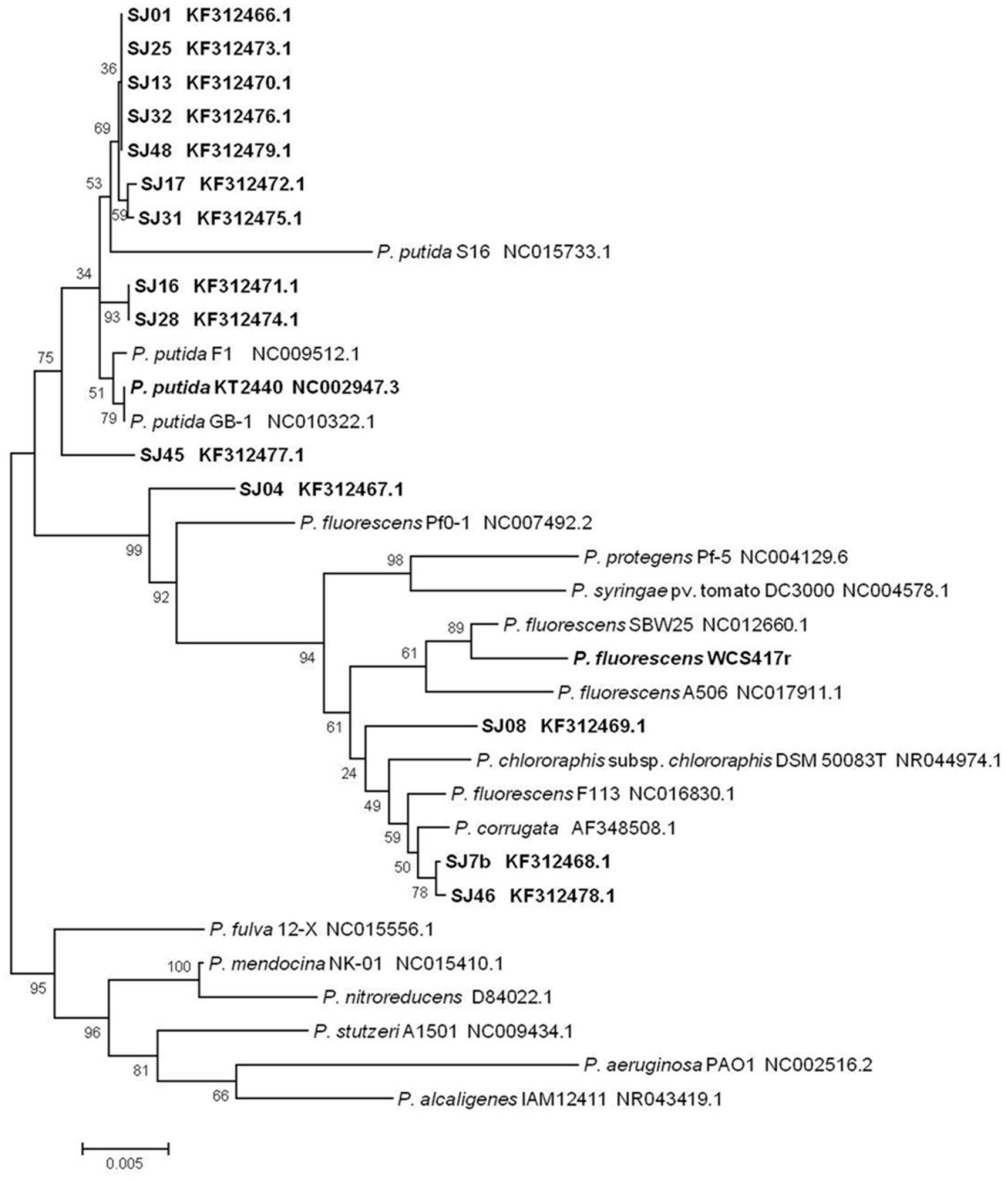
**Phylogenetic tree of native and reference strains of fluorescent ***Pseudomonas*** based on aligned ***16S rRNA*** sequences**. Native strains isolated from the *M. piperita* rhizosphere are indicated by boldface. The tree was constructed based on multiple alignments and the neighbor-joining method, using the MEGA 4.0.2 software program. Bootstrap values on branches are shown as percentages from 1000 replications. Scale bar at bottom represents 5 nt substitution per 1000 nt sequences. GenBank accession numbers are shown.

A phylogenetic tree was constructed to determine relationships and evolutionary distances among the fluorescent *Pseudomonas* strains isolated in this study, and the major, well-known species of this genus (Figure 3). We utilized the GenBank database because of its size and the ability to download sequences closely related to the fluorescent group (Altschul et al., 1997). Two major clusters appear in the phylogenetic tree: the first contains *P. putida, P. fluorescens, P. protegens, P. syringae, P. chlororaphis*, and *P. corrugate*; the second contains *P. fulva, P. mendocina, P. nitroreducens, P. stutzeri, P. aeruginosa*, and *P. alcaligenes*. The first cluster includes all the native fluorescent *Pseudomonas* strains isolated from the *M. piperita* rhizosphere, and can be divided into two subclusters centered on *P. putida* and *P. fluorescens*.

Most of the native strains analyzed had high similarity with *P. putida*. The strains most strongly related to *P. putida* were SJ16 and SJ28, followed by SJ01, SJ13, SJ17, SJ25, SJ31, SJ32, and SJ48. Strain SJ45 was distant from this group. These findings are consistent with genotypic and phenotypic analyses, and confirm that *P. putida* is the major fluorescent species present in the *M. piperita* rhizosphere.

The remaining four strains were part of the *P. fluorescens* subcluster and were notably distant from the *P. putida* subcluster, again consistent with results of genotypic and phenotypic analyses. Strain SJ04was intermediate in phylogenetic position between the two subclusters, and showed a phylogenetic relationship with *P. fluorescens* in spite of its identity with *P. putida* (Table 4). Strains SJ7b and SJ46 had a stronger phylogenetic relationship with *P. corrugate* than with *P. fluorescens*, consistent with results of BLAST analysis. Strain SJ08 had a phylogenetic relationship with *P. fluorescens* and *P. chlororaphis*.

#### ERIC-PCR

Of the 40 native fluorescent strains analyzed, only 38 generated an appropriate profile; SJ32 and SJ35 did not show amplification under the conditions applied. The primers used generated 67 markers with sizes ranging from 100 to 1700 bp, represented only by polymorphic bands. The pairs SJ21/SJ22, SJ17/SJ18, and SJ38/SJ39 showed identical fingerprinting (100% similarity); i.e., each of these pairs was likely the same strain.

**Figure 4:**
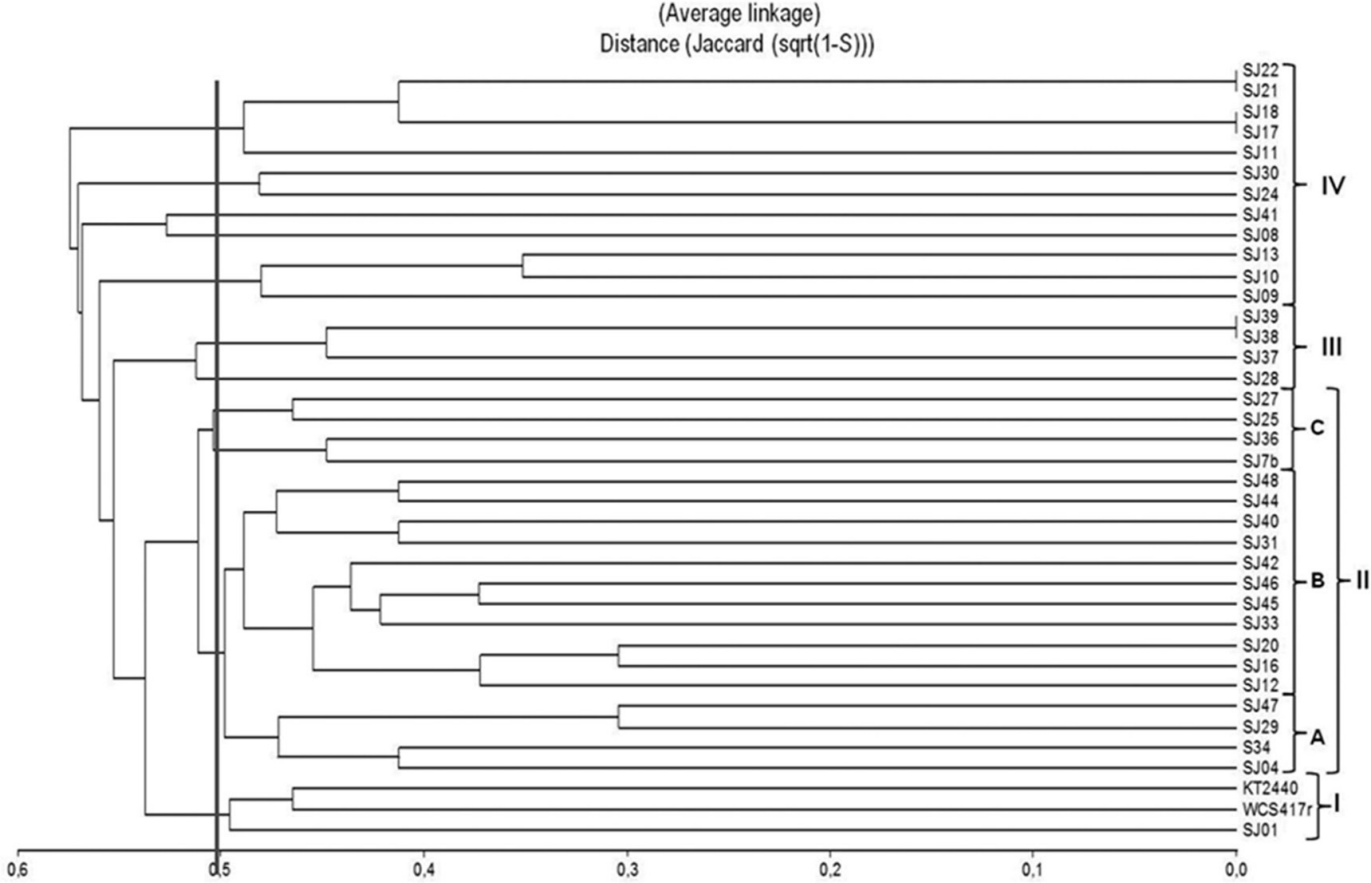
**Dendrogram based on ERIC-PCR profiles, generated from native, and reference ***Pseudomonas*** strains using the UPGMA algorithm and Jaccard coefficient**.

A dendrogram was constructed to determine genetic distance or proximity among the isolated strains (Figure 4). Clusters were generally observed at greater genetic distances than in ARDRA analysis, most likely because of the higher number of markers generated and their distribution along the whole genome. At 50% similarity, four groups (I-IV) were formed. The main group (II) included 19 strains and was divided into three subgroups (A-C). Within these subgroups, the most closely related strains were SJ16 and SJ20 (70% similarity), SJ29 and SJ47 (70% similarity), and strains SJ45 and SJ46 (65% similarity). Group I included reference strains *P. fluorescens*WCS417r and *P. Putida* KT2440 with 55% similarity. Groups III and IV were smaller than groups I and II.

ERIC-PCR analysis did not allow us to distinguish between the two fluorescent reference strains because they presented 50% similarity, the same as observed for various native strains (Figure 4). In previous studies, this technique was used to evaluate diversity/similarity within groups of native *Pseudomonas* strains, and gave comparable degrees of similarity (Achouak et al., 2000; Wolska and Szweda, 2008; Selvakumar et al., 2009; Vyas et al., 2009). In contrast, it did not give high degrees of similarity for native fluorescent *Pseudomonas* isolated from the *M. piperita* rhizosphere, suggesting that these populations have considerable genetic diversity in spite of their phylogenetic affiliation with *P. putida*.

#### BOX-PCR

Each of the 40 analyzed strains generated an appropriate profile using the primer BOXA1R. This technique allowed a high degree of discrimination among native fluorescent strains isolated from the *M. piperita* rhizosphere. It is frequently applied for grouping of *Pseudomonas* strains isolated from soil samples (Naik et al., 2008; Vyas et al., 2009; Mehri et al., 2011; Parejko et al., 2012). Of the 40 strains, 39 generated an appropriate profile, whereas strain SJ25 did not undergo amplification under the conditions applied. In comparison with ERIC-PCR, BOX-PCR analysis generated a larger number of markers. A total of 104 discrete polymorphic bands were scored, ranging in size from 250 to 2000 bp, with 8 to 20 bands for each strain. Six strains presented the same profile, and were presumably identical.

**Figure 5:**
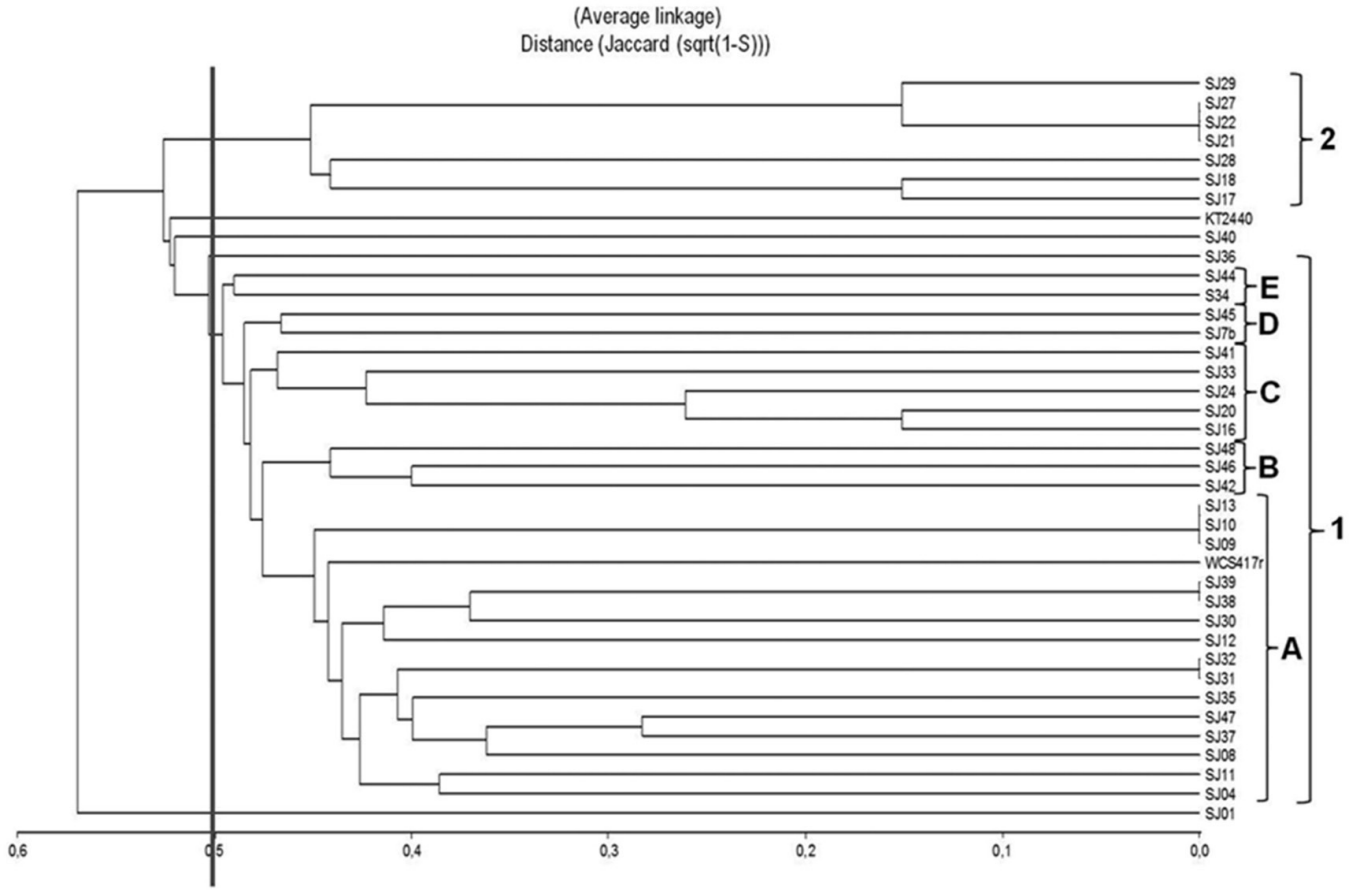
**Dendrogram based on BOX-PCR profiles, generated from native, and reference ***Pseudomonas*** strains using the UPGMA algorithm and Jaccard coefficient**.

A dendrogram was constructed to determine genetic distance or proximity among the isolated native strains (Figure 5). The BOX-PCR dendrogram was highly complex, and showed formation of a larger number of clusters at higher similarities. All strains grouped at 48% similarity, except for SJ01 which separated from the other strains at lower similarity. A large cluster (1) was formed at 50% similarity. It included the majority of strains (29), and was divided into five subclusters (A–E). The main subcluster (A), formed at ~55% similarity, included 15 of the native strains and reference strain *P. fluorescens* WCS417r. Within subcluster A, 100% similarity was obtained for three groups of strains: SJ09/SJ10/SJ13, SJ38/SJ39, and SJ31/SJ32. A smaller cluster (2) included high-similarity groups; e.g., SJ21/SJ22/SJ27 showed 100% similarity among themselves and 85% similarity with SJ29.SJ17/SJ18 grouped at 85% similarity. Reference strain *P. putida* KT2440 was not part of either cluster 1 or 2, and showed only low similarity (~55%) with cluster 1.

In contrast to ERIC-PCR, BOX-PCR analysis did not give high similarity levels among reference strain *P. putida* KT2440 and the native strains, but did give high similarity levels among reference strain *P. fluorescens* WCS417r and the native strains. These rep-PCR techniques, applied together, thus provide a valuable tool for evaluating diversity of native fluorescent *Pseudomonas* strains isolated from the *M. piperita* rhizosphere, and most likely from rhizospheres of other aromatic species as well.

#### Comparison of molecular techniques applied

In a comparison of the two rep-PCR techniques, ERIC-PCR showed better discrimination among native strains. The number of markers obtained was lower, resulting in relatively easy analysis and a high degree of clustering, i.e., lower similarity among native fluorescent *Pseudomonas* strains isolated from the *M. piperita* rhizosphere. At 80% similarity, 35 groups were obtained from ERIC-PCR analysis, vs. 30 groups obtained from BOX-PCR analysis. ERIC-PCR was useful for detailed study of differences in genomic DNA among closely related strains. BOX-PCR was useful for grouping strains while eliminating small differences among them, resulting in larger clusters. BOX-PCR produced a larger number (6) of duplicate profiles (Figure 4), i.e., a larger number of identical strains, than the number (3) obtained from ERIC-PCR (Figure 3). Previous studies gave similar degrees of heterogeneity by application of rep-PCR analysis in groups of isolated *Pseudomonas* strains (Selvakumar et al., 2009; Vyas et al., 2009; Wolska et al., 2011).

Simultaneous application of ARDRA and rep-PCR techniques could not distinguish among *P. fluorescens* and *P. putida* strains because of the 50% similarity with reference strains. However, both types of analysis revealed close relationships within the SJ09/SJ10/SJ13, SJ16/SJ20, and SJ31/SJ32 groups. The SJ17/SJ18, SJ21/SJ22, and SJ38/SJ39 groups showed high similarity in rep-PCR but lower similarity in ARDRA. Although BOX-PCR and ERIC-PCR sequences allow us to deeply probe genomic differences, they cannot be used as a tool to identify or determine phylogenetic positions of native fluorescent strains. Phylogenetic positioning is based on comparison of *16S rRNA* gene sequences. ARDRA identifies small differences among native strains by evaluating this particular gene of interest. The combination of ARDRA and *rDNA 16S* sequencing clearly identified *P. putida* as the major fluorescent species in *M. piperita* rhizospheric soil.

### Plant growth-promoting effects of mVOCs

#### Biomass

Plants were analyzed after 30 days incubation with mVOCs. A significant (*p* ≤ 0.05) effect by mVOCs from native fluorescent Pseudomonas was observed for shoot fresh weight, which was 3.0 to 4.5-fold higher than control value. Values for shoot ramification number, node number, leaf number, root length, and dry weight did not differ significantly from control values (*p* > 0.05; Table 6). In one of our previous studies (Santoro et al., 2011), mVOCs from reference strain P. fluorescensWCS417r similarly increased shoot fresh weight of micropropagated *M. piperita*, but had no effect on leaf number or ramification number. The increase of shoot fresh weight can be attributed to greater leaf and stem size, leading to increased aerial biomass. We also observed a similar effect in *Ocimum basilicum* (basil) plants exposed to mVOCs from *B. subtilis* (Banchio et al., 2009). Zhang et al. (2007) explained the effect of mVOC exposure on plant biomass by demonstrating that stimulated genes were associated with cell wall structure, allowing expansion and reduced rigidity. They also observed reduced mVOC exposure in leaves, leading to enhanced shoot development through stimulation of elongation, and cellular differentiation (Zhang et al., 2008).

**Table 6:** **Effects of mVOCs emitted by native fluorescent ***Pseudomonas*** strains on biomass parameters of micropropagated ***M. piperita*** plantlets**.

**Treatments**	**Root**				**Shoot**		
	**Length (cm)**	**Dry weight (mg)**	**Fresh weight (mg)**	**Length (cm)**	**Node number**	**Ramification number**	**Leaf number**
Control	5.36 ± 1.20^a^	2.21 ± 0.40^a^	113.70 ± 12.34^a^	4.02 ± 0.39^a^	6.38 ± 0.19^a^	1.07 ± 0.15^a^	19.19 ± 1.62^a^
SJ01	7.62 ± 1.25^a^	4.89 ± 1.95^a^	233.87 ± 29.47^a^	4.45 ± 0.31^a^	7.29 ± 0.49^a^	1.25 ± 0.14^a^	23.23 ± 2.40^a^
SJ04	9.17 ± 1.25^a^	3.34 ± 0.57^a^	277.36 ± 26.52^b^	4.78 ± 0.39^a^	7.89 ± 0.45^a^	1.80 ± 0.64^a^	29.23 ± 3.71^a^
SJ7b	8.66 ± 0.98^a^	3.70 ± 0.60^a^	386.02 ± 79.05^b^	5.18 ± 0.17^a^	8.48 ± 1.05^a^	2.40 ± 1.20^a^	49.87 ± 2.21^a^
SJ08	7.37 ± 1.11^a^	3.37 ± 0.60^a^	227.23 ± 27.03^a^	4.60 ± 0.04^a^	7.00 ± 0.58^a^	1.15 ± 0.08^a^	25.44 ± 1.89^a^
SJ10	7.05 ± 1.50^a^	3.01 ± 0.65^a^	301.36 ± 46.81^b^	4.83 ± 0.31^a^	7.44 ± 0.39^a^	1.38 ± 0.39^a^	29.52 ± 2.90^a^
SJ13	7.77 ± 1.96^a^	3.31 ± 0.19^a^	298.82 ± 66.07^b^	4.84 ± 0.18^a^	7.81 ± 0.45^a^	1.56 ± 0.51^a^	27.88 ± 5.10^a^
SJ22	8.19 ± 1.57^a^	3.95 ± 0.93^a^	258.86 ± 17.41^a^	4.52 ± 0.20^a^	7.77 ± 0.81^a^	0.95 ± 0.05^a^	25.40 ± 2.22^a^
SJ24	9.92 ± 1.92^b^	2.56 ± 0.49^a^	251.72 ± 65.77^a^	4.80 ± 0.70^a^	7.53 ± 0.68^a^	1.75 ± 0.60^a^	30.56 ± 7.15^a^
SJ25	9.16 ± 1.35^a^	4.01 ± 0.77^a^	326.18 ± 69.86^b^	5.01 ± 0.30^a^	7.60 ± 0.32^a^	2.20 ± 0.41^a^	32.41 ± 6.34^a^
SJ27	9.15 ± 1.67^a^	4.57 ± 1.02^a^	279.09 ± 4.77^b^	4.64 ± 0.41^a^	7.66 ± 0.20^a^	1.40 ± 0.28^a^	27.17 ± 0.52^a^
SJ28	10.05 ± 2.89^a^	7.69 ± 3.54^a^	321.17 ± 46.66^b^	5.37 ± 0.43^a^	8.45 ± 0.52^a^	2.00 ± 0.71^a^	32.20 ± 7.71^a^
SJ31	7.66 ± 1.49^a^	5.12 ± 1.33^a^	346.38 ± 19.12^a^	5.21 ± 0.51^a^	8.15 ± 0.30^a^	2.20 ± 0.57^a^	31.10 ± 3.87^a^
SJ33	6.61 ± 0.80^a^	3.53 ± 0.48^a^	253.79 ± 27.37^a^	5.15 ± 0.21^a^	7.40 ± 0.72^a^	1.53 ± 0.35^a^	28.35 ± 2.71^a^
SJ36	8.75 ± 1.73^a^	3.55 ± 0.74^a^	221.97 ± 22.50^a^	4.83 ± 0.41^a^	7.00 ± 0.20^a^	1.44 ± 0.16^a^	22.94 ± 1.23^a^
SJ38	9.50 ± 0.77^a^	4.45 ± 1.06^a^	268.52 ± 28.95^b^	5.28 ± 0.19^a^	8.11 ± 0.31^a^	1.77 ± 0.36^a^	27.69 ± 2.52^a^
SJ40	9.13 ± 1.94^a^	6.01 ± 2.66^a^	277.08 ± 46.29^b^	4.92 ± 0.31^a^	7.60 ± 0.88^a^	2.23 ± 0.93^a^	28.75 ± 4.63^a^
SJ41	6.55 ± 0.85^a^	3.60 ± 0.83^a^	228.41 ± 14.24^a^	3.98 ± 0.19^a^	7.68 ± 0.39^a^	0.93 ± 0.39^a^	22.85 ± 3.05^a^
SJ44	7.76 ± 0.89^a^	4.21 ± 0.79^a^	238.85 ± 26.46^a^	4.77 ± 0.42^a^	7.30 ± 0.28^a^	1.23 ± 0.39^a^	23.86 ± 1.19^a^
SJ45	9.37 ± 1.08^a^	3.79 ± 0.33^a^	261.12 ± 26.05^a^	4.99 ± 0.13^a^	7.86 ± 0.59^a^	1.16 ± 0.10^a^	26.19 ± 2.94^a^
SJ46	8.06 ± 0.60^a^	3.94 ± 0.70^a^	315.16 ± 62.22^b^	5.21 ± 0.34^a^	8.22 ± 0.68^a^	1.93 ± 0.40^a^	32.69 ± 4.16^a^
SJ48	8.23 ± 1.06^a^	4.75 ± 1.10^a^	265.52 ± 26.93^b^	5.06 ± 0.50^a^	8.03 ± 0.28^a^	1.41 ± 0.21^a^	26.44 ± 0.58^a^
WCS417r	6.91 ± 1.00^a^	3.62 ± 0.75^a^	262.45 ± 28.97^a^	4.66 ± 0.24^a^	7.10 ± 0.63^a^	1.61 ± 0.17^a^	26.03 ± 2.00^a^

a,b *Means followed by the same letter within a column are not significantly different according to Fisher's LSD test (p < 0.05)*.

#### EO production

We examined the effect of mVOCs on EO production in micropropagated *M. piperita* plantlets. The major components of extracted EOs were (−) limonene, terpineol, (−) menthone, (+) menthofuran, (−) menthol, (+) pulegone, and (−) menthyl acetate.

**Figure 6:**
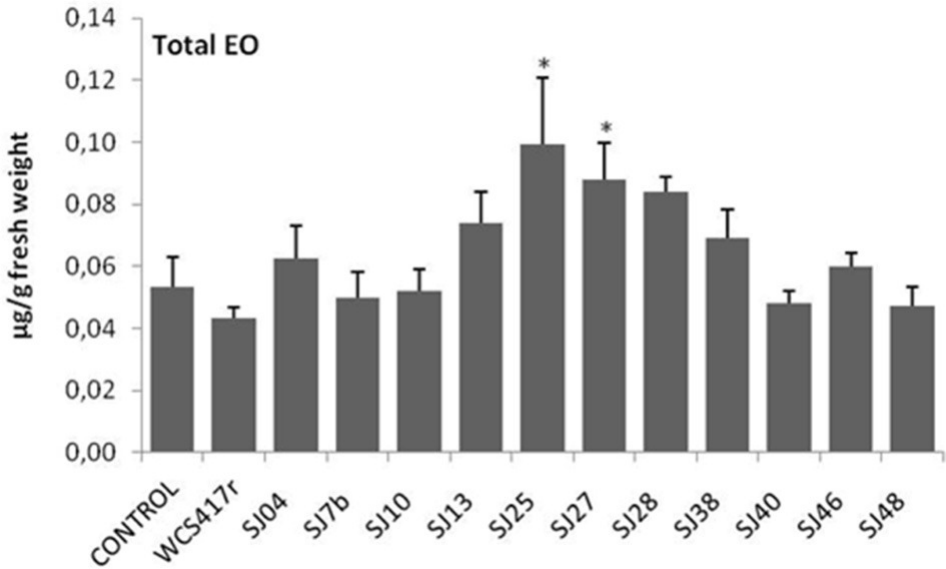
**Effects of exposure to mVOCs of native and reference strains of fluorescent ***Pseudomonas***on EO content of micropropagated ***M. piperita*** plantlets**. ^*^Significant difference (*p* ≤ 0.05) from control value.

**Figure 7:**
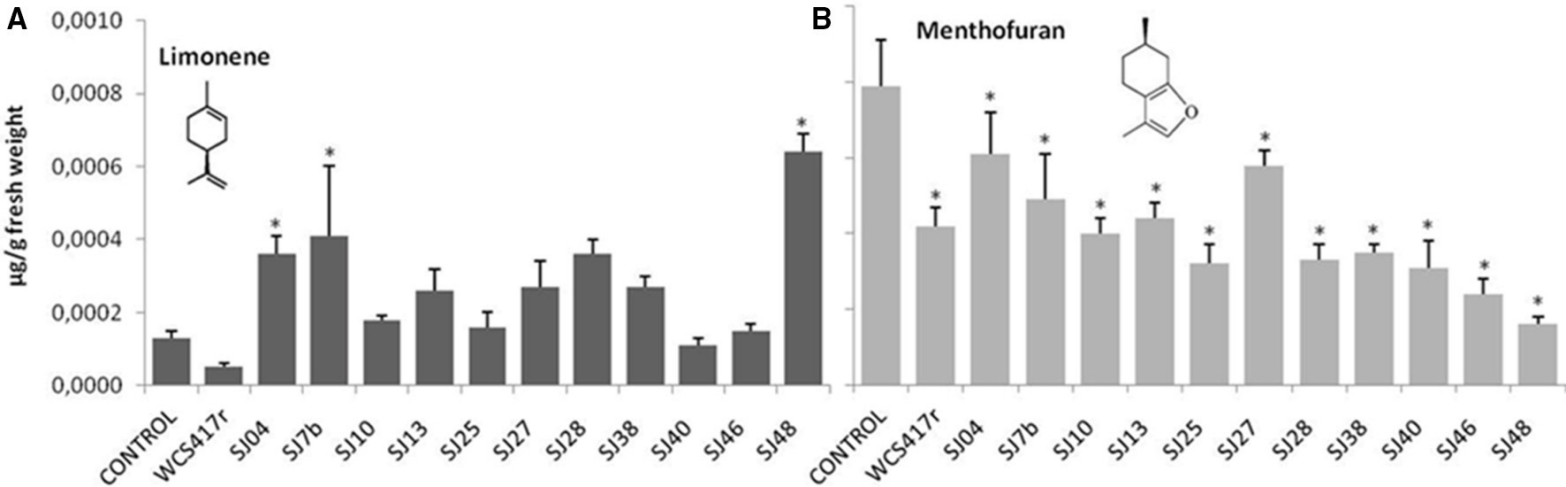
**Effects of exposure to mVOCs of native and reference strains of fluorescent ***Pseudomonas***on limonene and menthofuran content of micropropagated ***M. piperita*****. ^*^Significant difference (*p* ≤ 0.05) from control value.

**Figure 8:**
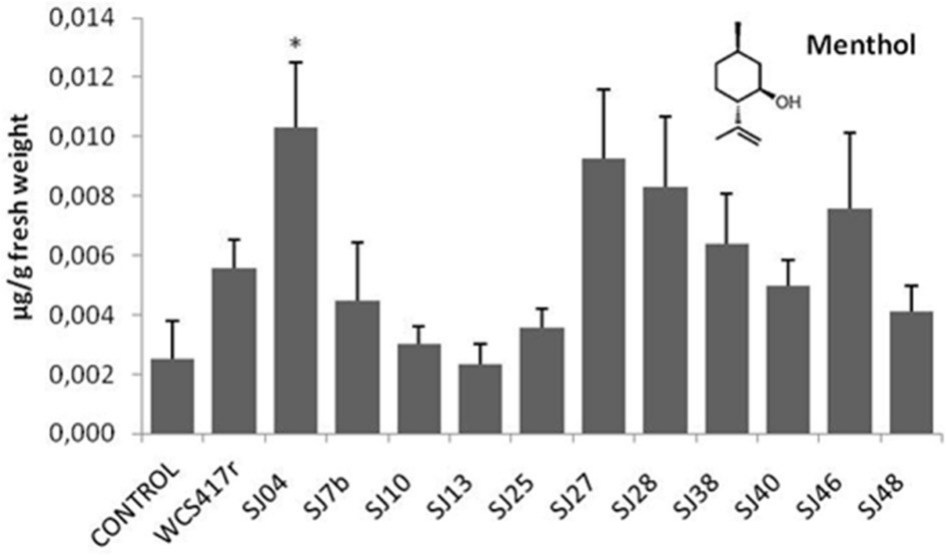
**Effects of exposure to mVOCs of native and reference strains of fluorescent ***Pseudomonas***on menthol content of micropropagated ***M. piperita*****. ^*^Significant difference (*p* ≤ 0.05) from control value.

**Figure 9:**
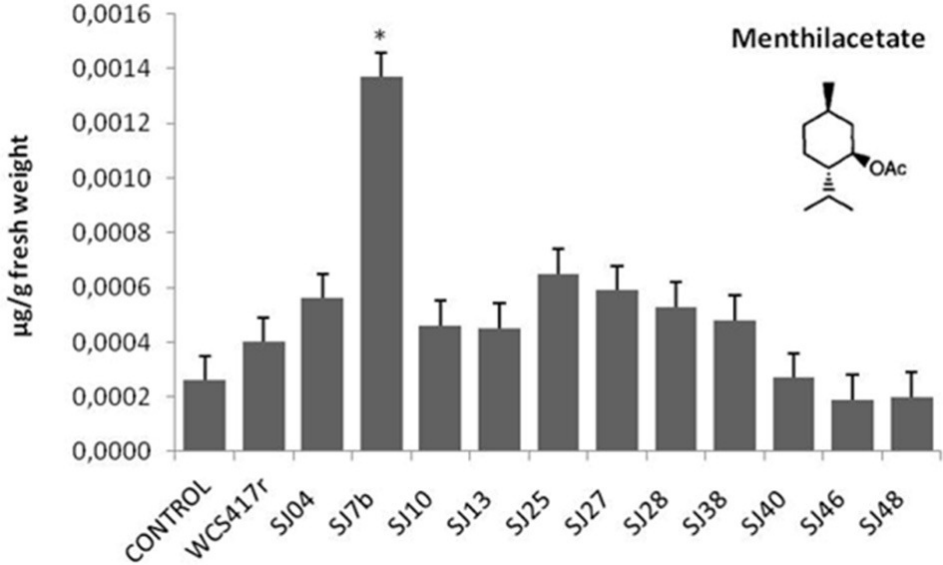
**Effects of exposure to mVOCs of native and reference strains of fluorescent ***Pseudomonas***on menthylacetate content of micropropagated ***M. piperita*****. ^*^Significant difference (*p* ≤ 0.05) from control value.

Exposure to mVOCs from strains SJ25 and SJ27 resulted in 86 and 57% increases, respectively, in total EO production relative to control (Figure 6). Limonene content was increased 2-fold by exposure to SJ04, SJ28, or SJ7b mVOCs, and 4-fold by SJ48mVOCs (Figure 7). Menthol content was increased three-fold by SJ04 mVOCs (Figure 8). Menthofuran Limonene content was reduced by exposure to mVOCs from all strains, most notably (20% reduction) by SJ48 mVOCs (Figure 7). Menthyl acetate content was increased four-fold by SJ7b mVOCs (Figure 9). The changes mentioned above were statistically significant at *p* ≤ 0.05. Changes in menthone, terpineol, and pulegone content produced by exposure to mVOCs were not significant (*p* > 0.05; data not shown).

Content of menthol, the major monoterpene, was significantly elevated only by SJ04 mVOCs. Monoterpenes are the commercially valuable components among EOs (Gupta et al., 2002). The observed reduction of menthofuran content is related to the increase in menthol, because menthofuran represents a branch in the flux of terpene precursors to final production of menthol (Lange and Croteau, 1999; Turner and Croteau, 2004). The increased levels caused by SJ04, SJ7b, and SJ48 mVOCs may reflect a lack of maturation of EOs, considering that enzymes involved in later reactions are activated at ~30 days of foliar growth (McConkey et al., 2000). Thus, our findings suggest that mVOCs produced by native fluorescent strains stimulate monoterpene synthesis in micropropagated *M. piperita*.

Enhanced production of secondary metabolites may depend directly on improved nutritional status and on primary metabolism of plants following PGPR inoculation (Shulka et al., 1992; Giri et al., 2003; Farag et al., 2006). Studies by Zhang et al. (2007, 2008, 2009) suggest that plants exposed to mVOCs experience high availability of nutrients for growth and increased level of photosynthesis. These phenomena could lead to improved distribution of energy to support synthesis of secondary metabolites such as monoterpenes. Alternatively, they could reflect defensive responses of plants to bacterial colonization following PGPR inoculation, in view of the known antimicrobial properties of EOs (Hammerschmidt and Nicholson, 1999; Sangwan et al., 2001; Wittstock and Gershenzon, 2002). Studies by Radruppa et al. (2010) demonstrated induced systemic resistance (ISR) mediated by ethylene and salicylic acid in *A. thaliana* exposed to acetoin, a component of mVOCs produced by *B. subtilis* (Farag et al., 2006). Even in the absence of contact between a plant and bacterial mVOCs, enhanced EO production resulting from improved nutritional status could be mediated by improved defensive ability of the plant following mVOC exposure.

### Chemical analysis of mVOCs

mVOCs of native strains SJ04, SJ25, and SJ48, and reference strain *P. fluorescens* WCS417r, were analyzed using Super Q® filters. Only compounds not present in control samples (Table 7) were considered. Strain SJ48 showed the greatest mVOC production. Strains SJ25 and WCS417r had the same chromatographic profile. A total of 11 components were identified, belonging to 3 classes: hydrocarbons (cyclohexane, decane, dodecane, 2,6,10-trimethyl-tetradecane, dotriacontane, 11-decyl-docosane), aromatic compounds (methyl-benzene, 2-benzyloxy-ethanamine, 1-methylnonadecyl-benzene, 1-(N-phenylcarbamyl)-2-morpholinocyclohexene), and halogenated compounds (1-chlorooctadecane). Hydrocarbons such as cyclohexane and dodecane were detected in strains of the genera *Bacillus* (Farag et al., 2006) and *Paenibacillus* (Lee et al., 2012), and were found to increase plant biomass in *A. thaliana*. They also induced ISR status, even though they were not the major mVOC components. The hydrocarbons decane and dodecane were detected in *Pseudomonas* strains, but did not display specific biocontrol activity against the plant pathogenic fungus *Sclerotinia sclerotiorum* (Fernando et al., 2005).

**Table 7:** **mVOCs identified in headspace extracts of native and reference strains of fluorescent ***Pseudomonas*****.

**rt (min)**	**Compound**	**Fluorescent** ***Pseudomonas*** **strains**			
		**SJ04**	**SJ25**	**SJ48**	**WCS417r**
6.20	Cyclohexane		xxx		xxx
7.92	2-(benzyloxy)ethanamine			x	
8.02	Benzene, methyl		xxx		xxx
12.30	Decane			x	
13.28	1-(N-phenylcarbamyl)-2-morpholinocyclohexene	xx			
14.21	Dodecane			x	
15.24	Benzene, (1-methylnonadecyl)			x	
15.95	1-chlorooctadecane			x	
17.15	Tetradecane, 2,6,10-trimethyl			x	
20.59	Dotriacontane			x	
22.79	Docosane, 11-decyl			x	

*Compounds were identified based on comparison of mass spectra with the NIST database. Chemical signals observed in both medium control and samples are not shown. Percentages of compounds are classified as 1–10% (x), 10–30 (xx), or 30–100% (xxx)*.

We conclude that the obtained chromatographic profiles are characteristic of the three native strains analyzed. This is interesting considering that the strains were isolated from the same location and presumably subjected to similar selective pressures.

It should be noted that low chemical diversity in a pool of mVOCs does not necessarily relfect low activity of the producer strain. Previous studies, even when focused on the major compound in the mVOC chemical profile, have consistently concluded that the degree of the observed effect reflects synergism among the compounds present. Even when investigators evaluated promoting activity using standards, they did not observe the same level of response in the parameters analyzed in comparison with the entire mVOC pool (Fernando et al., 2005; Farag et al., 2006; Radruppa et al., 2010; Groenhagen et al., 2013).

From a functional point of view, the various mVOC pools did not generate the same effects. Each of the native fluorescent bacterial strains analyzed had different PGPR activity. Although the strains all produced an increase in total fresh weight, their enhancing effect on EO content was variable, be causing of differing mixtures of emitted mVOCs. For example, mVOCs produced by strain SJ25 increased total EO content without improving the composition. In contrast, SJ04 and SJ48mVOCs improved the composition of EOs produced by the plants. Exposure of plants to SJ04 mVOCs resulted in decreased menthofuran and increased limonene and menthol contents. Exposure to SJ48 mVOCs similarly resulted in decreased menthofuran and increased limonene. These strains improved the quality of extracted EOs because of their ability to reduce menthofuran production, causing a diversion to the menthol production pathway, the primary indicator of EO quality (Lange and Croteau, 1999; Turner and Croteau, 2004). Clearly, differing mVOC pools emitted by bacteria induce differential responses in micropropagated *M. piperita*, and allow us to improve both quantity and quality of EO production.

## Conclusion

We identified *Pseudomonas putida* as the predominant fluorescent PGPR species present in rhizospheric soil of *Mentha piperita* (peppermint), using a combination of phenotypic testing and molecular techniques (ARDRA, *rDNA 16S* sequence analysis). This species accounted for ~60% of native strains analyzed. Other *Pseudomonas* strains present in the rhizospheric soil were also identified. The whole genome of the fluorescent bacterial population isolated was evaluated using rep-PCR techniques (ERIC-PCR, BOX-PCR), which revealed the overall complexity of the population and the degree of dissimilarity among strains.

This is the first study focused on fluorescent bacterial populations in rhizospheric soil of aromatic plants in general and *M. piperita* in particular. The isolated native bacterial strains promoted *M. piperita* growth, through mVOC production. Exposure of plants to mVOCs results in increased shoot fresh weight and EO production, through improved quality of EO composition. The obtained chromatographic profiles were characteristic of the three native strains analyzed, and each mVOC pool generated a different level of PGPR activity in micropropagated *M. piperita* plantlets, which is interesting considering that the strains were isolated from the same location and presumably subjected to similar selective pressures. Our findings provide a basis for extensive further studies, and demonstrate the importance of PGPR properties in analysis of isolated native strains, in terms of phylogenetic position and adaptation to environmental and edaphic conditions. The combined analytical approach used represents a significant biotechnological advance in understanding of growth promotion of aromatic plants by rhizobacteria.

## Author contributions

Conceived and designed the experiments: MS, EB. Performed the experiments: MS, PB, NN, LC. Analyzed the data: MS, PB, NN, LC, WG, EB. Contributed reagents/materials/analysis tools: MS, PB, NN, LC, WG, EB. Wrote the manuscript: MS, PB, EB.

### Conflict of interest statement

The authors declare that the research was conducted in the absence of any commercial or financial relationships that could be construed as a potential conflict of interest.
